# Aqueous Mulberry Leaf Extract Ameliorates Alcoholic Liver Injury Associating with Upregulation of Ethanol Metabolism and Suppression of Hepatic Lipogenesis

**DOI:** 10.1155/2021/6658422

**Published:** 2021-05-08

**Authors:** Yi-Ju Lee, Ming-Chang Tsai, Hui-Ting Lin, Chau-Jong Wang, Shao-Hsuan Kao

**Affiliations:** ^1^Department of Pathology, Chung Shan Medical University Hospital, Taichung 402, Taiwan; ^2^Department of Pathology, School of Medicine, Chung Shan Medical University, Taichung 402, Taiwan; ^3^School of Medicine, Chung Shan Medical University, Taichung 402, Taiwan; ^4^Division of Gastroenterology and Hepatology, Department of Internal Medicine, Chung Shan Medical University Hospital, Taichung 402, Taiwan; ^5^Institute of Medicine, Chung Shan Medical University, Taichung 402, Taiwan; ^6^Clinical Laboratory, Chung Shan Medical University Hospital, Taichung 402, Taiwan; ^7^Department of Health Diet and Industry Management, Chung Shan Medical University, Taichung 402, Taiwan

## Abstract

Excessive alcohol intake is a major cause of chronic liver damage and is highly associated with the development of a spectrum of hepatic disorders, including steatohepatitis, liver cirrhosis, and liver cancer. Thus, we aimed to explore the hepatoprotective effects of an aqueous mulberry leaf extract (AME) on alcoholic fatty liver disorder (AFLD) by using a mouse model fed with excessive ethanol. Compared with the normal diet, the ethanol diet significantly increased the body weight of the mice, while the AME supplement reduced the weight gain caused by the ethanol diet. The ethanol diet also attenuated the activity of alcohol dehydrogenase and antioxidant enzymes but increased lipid peroxidation in the liver, which were reversed by AME supplementation. Additionally, AME supplementation diminished the ethanol diet-induced hepatic leukocyte infiltration and expressions of IL-6 and TNF*α*. Moreover, AME supplementation also reduced the ethanol-diet-induced lipid accumulation and expression of 1-acylglycerol-3-phosphate acyltransferase, acetyl-CoA carboxylase, low-density lipoprotein receptor, and sterol regulatory element-binding protein-1/2 in the liver. Collectively, AME supplementation improved liver lipid accumulation and proinflammatory response in mice induced by the ethanol diet, which was associated with the upregulation of ethanol-metabolizing enzymes and the downregulation of lipogenesis components.

## 1. Introduction

Alcoholic fatty liver disorder (AFLD), including alcoholic fatty liver, steatohepatitis, liver fibrosis, liver cirrhosis, and hepatoma, is a leading cause of human death worldwide [1]. Excessive alcohol intake contributes to oxidative stress in the liver due to the accumulation of reactive oxygen species (ROS) associated with alcohol metabolism [2]. In addition, alcohol metabolites such as acetaldehyde lead to hepatic inflammation and other types of hepatotoxicity [3]. During the progression of AFLD, fatty liver, which is the accumulation of lipids in the liver, is the only condition that is considered to be reversible without drug intervention [4]. Therefore, the early diagnosis of AFLD and prevention of further development of fatty liver into advanced AFLD are critical in the treatment of AFLD.

Mounting evidence shows that nontoxic compounds extracted from natural foods and herbal plants can prevent AFLD [5]. Among the herbal plants, mulberry (*Morus alba* L.) leaf contains abundant polyphenols such as caffeic acid, gallate, and quercetin [6] and are traditionally used as a medicinal herb to ameliorate metabolic syndromes such as dyslipidemia, fatty liver, hypertension, and diabetes [7–9]. Previous studies indicated that mulberry leaf extract helps improve the obesity-induced fatty liver by regulating hepatic lipid metabolism, inhibiting fibrosis, and promoting antioxidant system [10]. However, it remains unclear whether or not mulberry leaves have beneficial effects on AFLD.

The aim of this study was to identify the protective effects of aqueous mulberry leaf extract (AME) on alcoholic fatty liver focusing on oxidative stress, inflammation, ethanol metabolism, and hepatic lipogenesis by using an *in vivo* animal model. Our findings revealed that AME clearly upregulated ethanol metabolic enzyme activities and antioxidant capability, reduced proinflammatory response, and inhibited hepatic lipogenesis. Thus, we suggest that AME may be beneficial to improve alcohol-induced liver injury.

## 2. Materials and Methods

### 2.1. Preparation of AME and Composition Analysis

The extraction was performed as previously described with some modification^11^. Briefly, fresh mulberry leaves (100 g) were harvested, air-dried at 50°C, homogenized by using a blender, and then, incubated in 1500 mL deionized water for 8 hours (*h*). After the incubation, the residue was removed by filtration and the supernatant was collected, frozen at −80°C, and then, lyophilized. The lyophilized powder was used as AME for the subsequent experiments. Composition analysis was performed as previously described [11], and the results showed that the polyphenol components of AME consisted of neochlorogenic acid (nCGA, 35.5%), cryptochlorogenic acid (cCGA, 31.7%), chlorogenic acid (CGA, 23.8%), rutin (9.2%), isoquercitrin (5.6%), astragalin acid (5.3%), nicotiflorin (3.5%), and protocatechuic acid (1.3%).

### 2.2. Animals and Liquid Alcohol Diets

Male C57BL/6J (B6) mice weighing 22 ± 2 g were purchased from the National Laboratory Animal Center of Taiwan (Taipei City, Taiwan) and kept under the supervision of the Institutional Animal Care and Use Committee (IACUC) of Chung Shan Medical University (IACUC-CSMU). All experimental protocols were made in accordance with the “Animal Protect Act” and “Guideline for the Care and Use of Laboratory Animals” and were approved on by the IACUC-CSMU (permit no. 1071, Dec 15, 2019), and all the animal experiments were carried out according to the approved protocols. After a 2-week maintenance for adaptation to the environment, a total of 40 mice were randomly divided into five groups by body weight (BW). Two mice were hosted in one cage separated by a transparent plastic plate, and each mouse had its own diet and water supply. Each group of mice received a unique diet for 10 weeks and weighed every two weeks. The five groups and their corresponding diets were (1) control, normal diet; (2) E, ethanol diet (Lieber–DeCarli liquid ethanol diet, positive control); (3) *E* + 0.5% AME, the ethanol diet containing 0.5% AME; (4) *E* + 1.0% AME, the ethanol diet containing 1% AME; and (5) *E* + 2.0% AME, the ethanol diet containing 2% AME. After feeding on different diets for 8 weeks, the mice were fasted for 12 h and then sacrificed to collect whole blood and liver tissue samples for the subsequent analysis.

### 2.3. Assessment of Serum Biomarkers

The assessment of liver function was performed by determining specific serum biomarkers, including aspartate transaminase (AST), alanine transaminase (ALT), total cholesterol (CHO), triglycerides (TG), alkaline phosphatase (ALP), high-density lipoprotein-cholesterol (HDL), and low-density lipoprotein-cholesterol (LDL), were conducted by using clinical chemistry reagent kits (Randox Laboratories Ltd., Antrim, UK) according to the manufacturer's institutions. Plasma samples were acquired by collecting blood using EDTA tubes and then centrifuged at 1500 x g for 10 min at 4°C. The resulting supernatant was transferred into a new tube and used as a plasma sample. Determination of biochemical factors in plasma was conducted by enzyme-coupling reactions and colorimetric measurement using an automatic analyzer (Olympus AU2700, Olympus Co., Tokyo, Japan).

### 2.4. Lipid Extraction and Quantitation for Liver Tissue

Liver lipids were extracted as previously described with some modification [12]. Briefly, the liver sample (1.25 g) was homogenized with a mixture of chloroform/methanol (1:2, 3.75 ml), and then, chloroform (1.25 mL) and distilled water (1.25 mL) were added to the homogenate and mixed well. After centrifugation at 1500 x g for 10 min, the upper supernatant was removed, and the lower clear organic solution was transferred into a new glass tube and lyophilized. After lyophilization, the dried powder was dissolved in chloroform/methanol (1 : 2) as the liver lipid extract and used for quantitation of hepatic TG and CHO.

### 2.5. Lipid Peroxidation Analysis for Liver Tissue

Lipid peroxidation was assessed by determining the level of thiobarituric acid-reactive substances (TBARS) as previously described [13]. Briefly, 200 *μ*L liver homogenate was mixed with 250 *μ*L of 25% (w/v) trichloroacetic acid, and then, the mixture was centrifuged at 10,000 x g for 30 min at 10^o^C. The resulting supernatant was collected and reacted with an equal volume of 1% thiobarbituric acid (TBA) for 40 min at 95°C in the dark for the formation of TBARS. After cooled at room temperature for 15 min, quantitation of TBARS was performed by using a Hitachi F2000 fluorescence spectrophotometer (excitation at 532 nm and emission at 600 nm) and 1, 1, 3, 3-tetraethoxypropane (TEP) standard curve (0–50 nM, *r*^2^ = 0.9951).

### 2.6. Histopathological Examination

Histological analysis of hepatic tissues was conducted as previously described [14]. Briefly, liver samples were fixed in 10% buffered neutral formalin, embedded in paraffin, cut into sections at a thickness of 3–5 *μ*m, and then, stained with hematoxylin and eosin. The histopathological changes including cell morphology and cellular lipid vesicles were examined by light microscopy (200X).

### 2.7. Protein extraction for Liver Tissue

The liver tissue (0.5 g) was homogenized with 5 mL radioimmunoprecipitation assay buffer (RIPA) buffer (Merck) containing protease inhibitors at 4°C using a Dounce homogenizer; then, the tissue homogenates were centrifuged at 10,000 x g for 20 min at 4°C to remove insoluble precipitate. The resulting supernatants (whole-tissue extracts) were used for the subsequent enzyme activity assay and western blot analysis. Protein concentrations of the whole-tissue extracts were determined using the Bradford protein assay kit (Bio-Rad, Hercules, CA, USA).

### 2.8. Western Blot

Crude proteins (30 *μ*g) were separated by sodium dodecyl sulfate-polyacrylamide gel electrophoresis (SDS-PAGE) and then transferred onto a polyvinylidene difluoride (PVDF) membrane (Millipore, Bedford, MA, USA). After blocked with 5% nonfat milk, the membrane was incubated with the primary antibody at 4°C for 16 h and then washed with phosphate-buffered saline containing 0.5% Tween-20. The bound primary antibodies were detected by secondary antibodies conjugated with peroxidase, and the antibody complex was visualized using a chemiluminescence substrate. Primary antibodies against human sterol regulatory element-binding protein 1 (SREBP-1, sc-13551), acetyl-CoA carboxylase (ACC, sc-137104), *β*-actin (sc-81178), and secondary antibodies were purchased from Santa Cruz Biotechnology (Santa Cruz, CA, USA). Antibody against 1-acylglycerolphosphate acyltransferase (AGPAT, ab67018) was obtained from Abcam, and chemiluminescence signals were detected and relatively quantitated by using the Fujifilm Las-3000 equipped with Multi Gauge software version 2.2 (Tokyo, Japan).

### 2.9. Activity Assay for Antioxidant Enzymes

Enzymatic activity assay for antioxidant enzymes was performed as previously described [15]. Briefly, the superoxide dismutase (SOD) activity was determined by a modified Marklund method, catalase activity was measured by the modified method proposed by Abei, and glutathione peroxidase (GSH-Px) activity was determined by the method of Lawrence and Burk.

### 2.10. Statistical Analysis

The quantitative data were presented as the mean ± SD from three independent experiments. The one-way ANOVA was used to analyze the significance of the difference using SPSS version 12 software. The difference with *P* < 0.05 was considered as statistically significant.

## 3. Results and Discussions

### 3.1. AME Supplementation Attenuated the Increased Body Weight (BW) of Mice and Serum Biomarkers in Response to Ethanol Feeding

**Figure 1:**
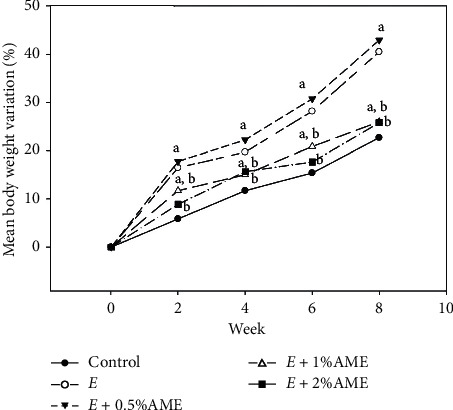
Body weight changes in mice fed with the ethanol diet and ethanol diet supplemented with AME. Mice were randomly divided into five groups (*n* = 8 for each group), namely, the control group (normal diet), E group (ethanol diet, the positive control), *E* + 0.5% AME group (ethanol diet containing 0.5% AME), *E* + 1% AME group (ethanol diet containing 1% AME), and *E* + 2% AME group (ethanol diet containing 2% AME). Body weight (BW) variation was presented as [(mean BW at week N–mean BW at week 0)/mean BW at week 0] x 100%. (a) *P* < 0.05 compared to the control group; (b) *P* < 0.01 compared to the E group.

We first explored the effects of AME supplements on changes in BW and serum biomarkers in response to ethanol feeding. As shown in Figure 1, after the 8-week administration of different diets, the BW of mice fed an ethanol diet clearly increased in a dose-dependent manner compared to that of mice fed the control diet (*P* < 0.05). At the end of week 8, the average BW of mice fed the ethanol diet was 1.41 times that of those fed the control diet. Compared to the ethanol diet, the ethanol diet supplemented with 1.0% or 2.0% AME significantly attenuated the increase in BW during the 8-week administration (*P* < 0.05, Figure 1). In contrast, 0.5% AME supplementation did not influence the increase in BW in response to ethanol feeding.

For the assessment of liver function, serum biomarkers in mice fed with different diets were determined. Compared to the control diet, the ethanol diet clearly elevated serum AST, ALT, CHO, TG, ALP, and LDL, but reduced serum HDL (Table 1). In addition,the ethanol diet supplemented with AME significantly reduced serum AST, ALT, CHO, TG, and LDL compared to the ethanol diet alone. However, AME supplementation insignificantly altered serum ALP and HDL compared to ethanol alone. Taken together, these observations showed that AME supplementation clearly attenuated the BW increase in mice and reversed the changes in serum biomarkers in response to ethanol feeding.

Collectively, our observations showed that alcohol intake elevated AST, ALT, and other liver function indicators, indicating that the AFLD model was properly induced as compared to previous studies [16, 17]. In addition, our results are also consistent with the findings that elevated TG and TC are associated with liver steatosis [18]. These observations indicate that AME supplementation can attenuate the BW increase in mice and reverse the changes in serum hepatic biomarkers in response to ethanol feeding, suggesting that AME may ameliorate alcoholic fatty liver injury.

### 3.2. AME Supplementation Enhanced the Activities of Hepatic ADH and ALDH in Ethanol-Fed Mice

**Figure 2:**
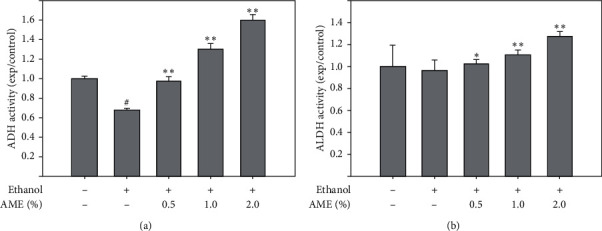
AME supplementation upregulated the activities of ALDH and ADH in the liver. Mice were randomly divided into five groups (*n* = 8 for each group), namely, the control group (normal diet), ethanol group (ethanol diet, the positive control), *E *+ 0.5% AME group (ethanol diet containing 0.5% AME), *E *+ 1% MLE group (ethanol diet containing 1% AME), and *E *+ 2% AME group (ethanol diet containing 2% AME). After 8-week administration, mice were fasted and sacrificed for the collection of liver tissues to assess activities of ADH and ALDH in the liver. Quantitative data are presented as means ± SD. ^#^, *P* < 0.05 compared to the control group; ^*∗*^ and ^*∗∗*^, *P* < 0.05 and *P* < 0.01 compared to the ethanol group, respectively.

Alcohol is metabolized to acetaldehyde and then to acetic acid by ADH and ALDH in the liver. Therefore, we examined whether or not AME regulated the enzymatic activities of hepatic ADH and ALDH. As shown in Figure 2, excess ethanol uptake clearly reduced the activity of hepatic ADH to 67.5 ± 0.8% of the control (*P* < 0.05), but did not significantly affect the activity of hepatic ALDH. In addition, AME supplementation dose-dependently increased the activities of hepatic ADH and ALDH up to 1.58 ± 0.48 and 1.27 ± 0.18 times, respectively, compared to that of the control (*P* < 0.01). These findings showed that AME supplementation increased the activities of ADH and ALDH in the livers of ethanol-fed mice, suggesting that AME may accelerate alcohol metabolism.

Alcohol is primarily metabolized by the ADH pathway at low concentrations, and the other metabolic systems, cytochrome P450 2E1 (CYP2E1) enzymes and catalase endoplasmic reticulum (CAT), begin to synergistically metabolize alcohol when the alcohol concentrations in blood and tissue fluid are higher than 10 mM [19]. Accordingly, our findings show that AME supplementation can increase the activities of ADH and ALDH in the livers of ethanol-fed mice, suggesting that AME may accelerate alcohol metabolism and, therefore, reduce the alcohol-induced liver injury.

### 3.3. AME Supplementation Upregulated Hepatic Antioxidant Enzymes and Reduced Hepatic Lipid Peroxidation in Mice Fed the Ethanol Diet

**Figure 3:**
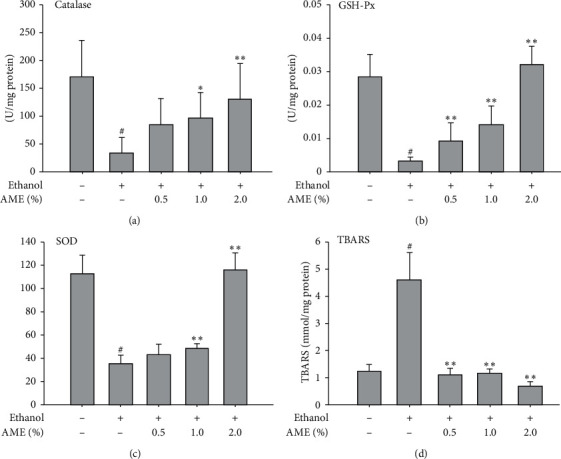
AME supplementation upregulated the activities of antioxidant enzymes and reduced the lipid peroxidation in the liver. Mice were randomly divided into five groups (*n* = 8 for each group), namely, the control group (normal diet), ethanol group (ethanol diet, the positive control), *E *+ 0.5% AME group (ethanol diet containing 0.5% AME), *E *+ 1% MLE group (ethanol diet containing 1% AME), and *E* + 2% AME group (ethanol diet containing 2% AME). After 8-week administration, mice were fasted and sacrificed for the collection of hepatic tissues to assess activities of catalase, glutathione peroxidase (GSH-Px), and superoxide dismutase (SOD) and level of thiobarbituric acid-reactive substances (TBARS) in the liver. Quantitative data were presented as means ± SD. #, *P* < 0.05 compared with the control group; ^*∗*^ and ^*∗∗*^, *P* < 0.05 and *P* < 0.01 compared to the ethanol group, respectively.

Oxidative stress is a major cause of hepatocyte injury; therefore, the effects of AME on antioxidant enzyme activity and lipid peroxidation in the liver were explored. As shown in Figure 3(a)–3(c), the activities of major antioxidant enzymes, catalase, SOD, and glutathione peroxidase (GSH-Px), were clearly attenuated in the livers of mice fed the ethanol diet compared to that of mice fed a normal diet (control). In contrast, AME supplements (1.0% and 2.0%) significantly restored the activities of catalase, SOD, and GSH-Px (*P* < 0.05, compared to the ethanol diet). Parallel to the attenuation of antioxidant activity, ethanol uptake also induced oxidative stress in the liver. As shown in Figure 3(d), hepatic TBARS levels were elevated in the liver of mice fed the ethanol diet compared to those of mice fed with a normal diet (Control, *P* < 0.05), and AME supplementation reduced the TBARS level in the liver of mice fed with the ethanol diet. These findings revealed that AME upregulated the activity of hepatic antioxidant enzymes and reduced hepatic lipid peroxidation in mice with a high-dose uptake of ethanol.

Mitochondrial dysfunction, lipid peroxidation elevation, and hepatic antioxidant reduction have been commonly recognized as the primary characters of AFLD [20]. Impairment of the antioxidative system such as SOD and GSH-Px [44] may further lead to cellular damage in response to a high-dose intake of ethanol [21]. In addition, downregulation of SOD level increases oxidative stress and initiates various pathological progresses *in vivo* [22]. Our findings revealed that AME upregulates the activity of hepatic antioxidant enzymes and reduced hepatic lipid peroxidation in mice with a high-dose uptake of ethanol, suggesting that AME supplementation may alleviate the progression of AFLD.

### 3.4. AME Supplementation Alleviated a Hepatic Inflammatory Response in Ethanol-Fed Mice

**Figure 4:**
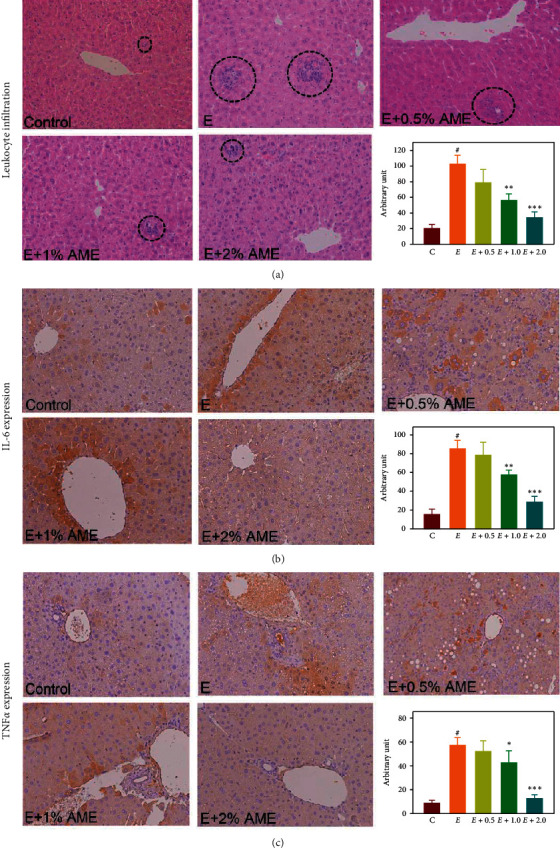
AME supplementation attenuated infiltration of leukocytes and expressions of proinflammatory cytokine IL-6 and TNF*α* in the liver. Mice were randomly divided into five groups (*n* = 8 for each group), namely, the control group (normal diet), ethanol group (ethanol diet, the positive control), *E *+ 0.5% AME group (ethanol diet containing 0.5% AME), *E* + 1% AME group (ethanol diet containing 1% AME), and *E *+ 2% AME group (ethanol diet containing 2% AME). After 8-week administration, mice were fasted and sacrificed for the collection of hepatic tissues for (A) histological examination by hematoxylin and eosin staining and (B, C) expression levels of hepatic IL-6 and TNF*α* by IHC staining.

Oxidative stress is highly associated with the induction of inflammation. Therefore, whether or not AME alleviates hepatic inflammation in response to excess ethanol uptake is investigated. As shown in Figure 4(a), leukocyte infiltration in the liver was significantly increased in mice fed with the ethanol diet compared to that of mice fed with a normal diet (Control), and the increased leukocyte infiltration was attenuated by AME supplements. Similarly, the expressions of inflammatory cytokines IL-6 and TNF*α* in the liver were clearly upregulated in response to excess ethanol uptake, and upregulations of IL-6 and TNF*α* were significantly lowered by AME supplementation (Figures 4(b) and 4(c)). Collectively, these results indicated that AME alleviated the ethanol-induced hepatic inflammatory response in mice.

TNF-*α* is an important inflammatory cytokine, which activates T cells and macrophages and induces the production of other inflammatory cytokines [23]. IL-6 is a multiple functional cytokine that plays a central role in the development of AFLD [24]. Our results show that AME can inhibit leukocyte infiltration and production of TNF*α* and IL-6 in the liver of mice fed on ethanol, indicating that AME may have an anti-inflammatory activity to alleviate the ethanol-induced hepatic inflammatory response and the consequent liver injury.

### 3.5. AME Supplementation Alleviated Macrovascular Steatosis and Decreased the Hepatic Lipid Content in Mice Fed with the Ethanol Diet

**Figure 5:**
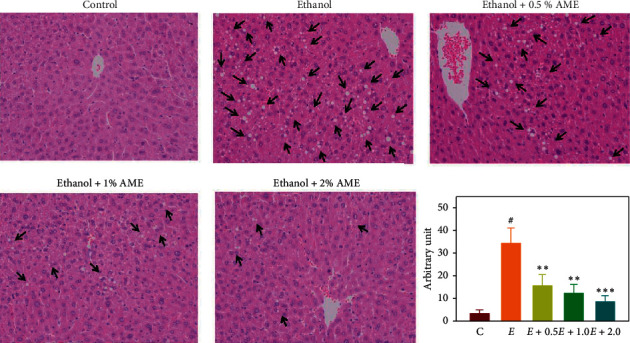
AME supplementation reduced accumulation of hepatic lipids in mice fed with the ethanol diet. Mice were randomly divided into five groups (*n* = 8 for each group), namely, the control group (normal diet), ethanol group (ethanol diet, the positive control), *E *+ 0.5% AME group (ethanol diet containing 0.5% AME), *E *+ 1% AME group (ethanol diet containing 1% AME), and *E* + 2% AME group (ethanol diet containing 2% AME). After 8-week administration, mice were fasted and sacrificed for the collection of liver tissues for histological change by hematoxylin and eosin staining. Hepatic lipid vesicles are indicated with arrows.

Heavy drinking induces the development of steatosis and lipid accumulation in the liver [25]. As a result, the effects of AME supplementation on the hepatic lipid content and steatosis were investigated. As shown in Figure 5, hematoxylin and eosin staining showed that macrovascular steatosis, a single large intracytoplasmic fat droplet in liver tissues, in mice fed with ethanol (*E*) was increased compared to that in mice fed with a normal diet (control). In contrast, AME supplements reduced macrovascular steatosis in the liver in a dose-dependent fashion. In addition to the histological examination for steatosis, the hepatic lipid contents were analyzed. As shown in Table 2, the contents of hepatic triglyceride and cholesterol in mice fed with the ethanol diet were significantly increased compared to those in mice fed with a normal diet (Control, *P* < 0.01), and the increased contents of hepatic triglycerides and cholesterol were dose-dependently reduced in mice fed with ethanol diet supplemented with AME (*P* < 0.01). Collectively, these observations showed that AME supplementation clearly reduced macrovascular steatosis in the liver and increased hepatic triglyceride and cholesterol contents in response to the ethanol diet.

Three major phenolic compounds, nCGA, cCGA, and CGA, have been identified in the mulberry leaf extract [11]. A previous study shows that nCGA and cCGA inhibit the production of IL-1*β*, IL-6, and TNF*α* by macrophages and reduces the cellular reactive oxygen species via upregulation of the Nrf2/HO-1 signaling [26, 27]. In addition, chlorogenic acid can reduce hepatic inflammation, hepatic lipid accumulation, and plasma hepatic enzyme activities in obese rats, but it does not change the plasma lipid profile [28]. Similarly, our findings reveal that AME upregulates antioxidant enzyme activities, reduces production of IL-6 and TNF*α*, and inhibits leukocyte infiltration in the liver, as well as decreases serum hepatic biomarkers and blood lipids in ethanol-fed mice. Thus, we suggest that AME, containing the three chlorogenic acid derivatives, has the combined effect of ameliorating ethanol-induced liver injury.

### 3.6. AME Supplementation Downregulated Triglyceride- and Cholesterol-Synthesis-Associated Mediators in the Liver of Ethanol-Fed Mice

**Figure 6:**
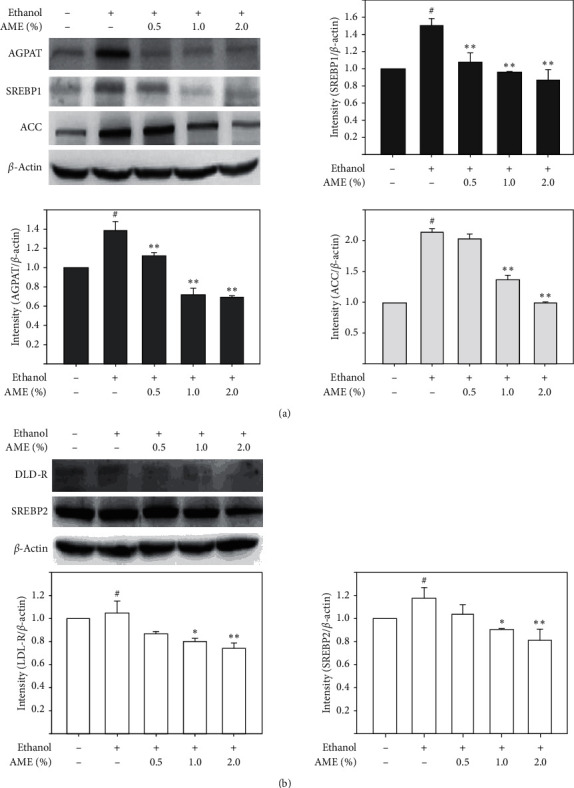
AME supplementation downregulated expressions of important mediators involved in fatty acid and cholesterol synthesis in the liver. Mice were randomly divided into five groups (*n* = 8 for each group), namely, the control group (normal diet), ethanol group (ethanol diet, the positive control), *E *+ 0.5% AME group (ethanol diet containing 0.5% AME), *E *+ 1% AME group (ethanol diet containing 1% AME), and *E *+ 2% AME group (ethanol diet containing 2% AME). After 8-week administration, mice were fasted and sacrificed for the collection of hepatic tissues for protein extraction and western blot analysis. Quantitative analysis of chemiluminescence signals from three repeated western blot analyses is presented as means ± SD. ^#^, *P* < 0.05 compared to the control group; ^*∗*^ and ^*∗*^, *P* < 0.05 and *P* < 0.01 compared to the ethanol group, respectively.

Since the ethanol-promoted hepatic lipid content was clearly reduced by AME supplementation, the effects of AME on triglyceride and cholesterol synthesis in the liver of mice fed with ethanol were explored. As shown in Figure 6(a), ethanol uptake clearly upregulated the expressions of acylglycerolphosphate acyltransferase (AGPAT), sterol regulatory element-binding protein 1 (SREBP1), and acetyl-CoA carboxylase (ACC) up to 1.47 ± 0.2, 1.5 ± 0.1, and 2.1 ± 0.1 times in the liver compared to those in the control (*P* < 0.05). In contrast, AME supplementation clearly reduced the upregulations of hepatic AGPAT, SREBP1, and ACC in a dose-dependent manner (*P* < 0.01). Similarly, ethanol uptake upregulated the expressions of low-density lipoprotein receptor (LDL-R) and SREBP2 in the liver, and the upregulation of LDL-R and SREBP2 in the liver was significantly reduced by AME supplementation (Figure 6(b), *P*) < 0.01). Taken together, it can be concluded that AME decreased the upregulations of triglyceride and cholesterol synthesis-associated mediators in the liver of mice fed with ethanol.

Excessive alcohol consumption not only induces hepatic inflammation and oxidative stress but also disrupts liver function, leading to dysregulation of hepatic lipid metabolism, upregulation of oxidative stress, and consequent liver damage and dyslipidemia [29]. Hepatic ACC, AGPAT, and LDL-R not only play an important role in liver lipid metabolism and antioxidant systems but are also involved in the development of alcoholic fatty liver [30]. Consistently, our findings showed that excess ethanol uptake increased the expression of AGPAT, LDL-R, SREBP1, and SREBP2 in the liver. Importantly, AME supplementation clearly restored the activity of these antioxidant enzymes and reduced the expressions of AGPAT, LDL-R, SREBP1, and SREBP2, indicating that AME may inhibit ethanol-induced triacylglycerol and cholesterol syntheses and attenuate the consequent fatty liver progression.

## 4. Conclusions

AFLD has been an important health issue in the modern society, which greatly worsens life quality and increases social burden. Our findings demonstrated that AME supplements had obvious beneficial effects on reducing liver damage and inflammatory response caused by excessive alcohol intake within 8 weeks, which may attribute to the promotion of alcohol metabolism and the attenuation of oxidative stress and inflammatory response in the liver.

## Figures and Tables

**Table 1:** Assessment of serum biomarkers in ethanol-fed mice supplemented with AME.

	Control	Ethanol	*E* + 0.5%AME	*E* + 1.0%AME	*E* + 2.0%AME
AST (U/L)	168.2 ± 41.7	463.5 ± 112.3^*##*^	340.2 ± 82.6^*∗*^	299.4 ± 65.3^*∗*^	265.4 ± 81.5^*∗∗*^
ALT (U/L)	35.6 ± 4.2	90.5 ± 10.45^*##*^	60.1 ± 15.6^*∗*^	55.4 ± 14.3^*∗*^	45.5 ± 7.7^*∗∗*^
CHO (mg/dL)	70.1 ± 7.6	88.2 ± 14.41	72.3 ± 6.7^*∗*^	68.1 ± 5.1^*∗*^	65.1 ± 3.02^*∗∗*^
TG (mg/dL)	80.2 ± 14.5	105.3 ± 8.8^*##*^	70.2 ± 11.52^*∗*^	65.1 ± 10.2^*∗∗*^	56.7 ± 7.8^*∗∗*^
ALP (mg/dL)	70.2 ± 9.63	109.2 ± 18.2^*##*^	100.6 ± 4.76	85.3 ± 11.4	80.3 ± 8.8
HDL (mg/dL)	40.8 ± 6.67	42.4 ± 5.3	41.2 ± 6.5	45.1 ± 15.5	38.00 ± 6.9
LDL (mg/dL)	25.3 ± 6.8	50.2 ± 6.9^*##*^	45.0 ± 4.8	44.2 ± 4.8	48.1 ± 4.9

Control, normal diet group; ethanol, ethanol diet group (the positive control); *E* + 0.5% AME, ethanol diet containing 0.5% AME group; *E *+* *1.0% AME, ethanol diet containing 1% AME group; *E *+* *2.0% AME, ethanol diet containing 2% AME group. Each value was expressed as the mean ± *S*.D (*n* = 8/group). Results were statistically analyzed with ANOVA. ^*##*^, *P* < 0.01 as compared to the normal group. ^*∗*^ and ^*∗∗*^, *P* < 0.05 and 0.01 as compared to the ethanol diet group (the positive control).

**Table 2:** Cholesterol and triglyceride content in the liver from ethanol-fed mice.

	Control	Ethanol	*E *+* *0.5%AME	*E *+* *1%AME	*E *+* *2%AME
TG (g/g protein)	63.60 ± 16.12	104.98 ± 34.71 ^*b*^	98.57 ± 21.85	61.44 ± 10.09^*c*^	58.55 ± 6.94^*c*^
CHO (g/g protein)	51.09 ± 20.00	106.50 ± 31.00 ^*b*^	38.57 ± 9.64	23.92 ± 13.31^*c*^	19.28 ± 7.20^*c*^

Control, normal diet group; E, ethanol diet group (the positive control); *E* + 0.5%AME, the ethanol diet containing 0.5% AME group; *E* *+* 1.0%AME, the ethanol diet containing 1% AME group; *E* + 2.0%AME, the ethanol diet containing 2% AME group. Each value was expressed as the mean ± *S*.D (8/group). *a* and *b*, *P* < 0.05 and 0.01 compared to the normal group, respectively. *c* and *d*, *P* < 0.05 and 0.01 compared to the ethanol diet group (the positive control), respectively.

## Data Availability

The datasets generated and/or analyzed in the present study are included in the manuscript.
